# Diseases of the Tear Passages

**Published:** 1908-01-11

**Authors:** A. Maitland Ramsay

**Affiliations:** Surgeon Ophthalmic Institution, and Consulting Ophthalmic Surgeon Royal Infirmary, Glasgow.


### HOSPITAL CLINICS.

### DISEASES OF THE TEAR PASSAGES.

By A. MAITLAND RAMSAY, M.D., F.F.P.S., Surgeon Ophthalmic Institution, and Consulting Ophthalmic Surgeon Royal Infirmary, Glasgow.

Abstract of a Post-graduate Lecture delivered at the Glasgow Ophthalmic Institution.

Before proceeding to investigate the causes and describe the methods of cure or relief of diseases of the tear-passages, it may be well to recur briefly to the anatomy and physiology of the lachrymal apparatus. The tears, coming from the lachrymal gland and mixed with secretions from the conjunctival glands, flow over the surface of the eyeball towards the inner canthus, where they collect in the hollow of the lacus lachrymalis, a space limited by the plica semilunaris on its outer side. Just at that point on the margin of both upper and lower eyelids where their direction changes and their clean-cut, flattened edge becomes rounded, there is a little papilla, the apex of which is turned towards the eyeball and perforated by a small orifice—the punctum lachrymale. The superior one is smaller than the inferior, and is so placed that when the eyelids are closed the puncta are not opposite, but lie side by side, the lower being external to the upper. The puncta lachrymalia lead into two minute ducts called the canaliculi, which usually unite to form a short tube, opening into the lachrymal sac about the junction of its upper fourth with the lower three-fourths. Each canaliculus is narrowest at the punctum, but immediately within the orifice the tube widens, the calibre diminishing again before the junction. The lachrymal sac is lodged in the groove formed by the lachrymal bone and the ascending process of the superior maxillary, and lies immediately behind the internal palpebral ligament. It opens into the nasal duct, a nearly cylindrical tube which passes downward, outward, and backward, to terminate in the inferior meatus of the nose.

The orbicularis palpebrarum, which has a very close relation to the canaliculi and the lachrymal sac, is divided into an orbital and a palpebral portion. Of these the latter alone acts in ordinary closing of the eyes, such as winking, while the former comes into play when the eyes are closed with some force. The precise mechanism for the conveyance of the tears into the lachrymal passages is not yet altogether understood, but it is certain that perfect closure of the lids is an indispensable condition. When the orbicularis contracts, the eyelids close, and the mouths of the canaliculi dip into the lacus lachrymalis and suck up the tears by capillary attraction. It is only when the lachrymal secretion is excessive, through over-stimulation of the gland or during alteration in the position of the puncta—as in laughing, coughing, vomiting, etc.—that the excretory channels become inadequate and tears overflow on to the cheeks.

Over-secretion usually affects both eyes, whereas blocking of the lachrymal ducts is, as a rule, unilateral. The chief causes of over-secretion are emotion and reflex disturbances, for example, neuralgia, a foreign body on the conjuctiva or cornea, affections of the nose or teeth, or optical defects.

By far the most common cause, however, of "watery eye" is displacement of the puncta or obstruction in the canaliculi. In all injuries involving the inner canthus—e.g. wounds, burns, etc.—the puncta, the canaliculi, and even the lachrymal sac, are very liable to be implicated, and after cicatrisation their function may be very seriously interfered with. The inferior canaliculus is the larger, and plays the more important part in excretion, so that when its punctum is displaced much lachrymation follows, even though the orifice of the superior duct is in accurate apposition to the eyeball. Displacement of the puncta may arise from want of tone in the fibres of the orbicularis palpebrarum, a condition frequently seen in the aged; or it may occur in paralysis of the facial nerve. Ectropion and entropion may take place from many different causes, and the punctum be thus withdrawn from the lacus lachrymalis.

A similar result may be brought about by the separation of the lid from the eyeball through chronic thickening of the palpebral conjunctiva, enlarged caruncle, or polypoid or other tumour of the conjunctiva situated near the inner canthus. The puncta are occasionlly congenitally absent, or they may be obliterated from disease of or injury to the eyelids. It rarely, however, happens that both upper and lower are destroyed, the latter being generally the one that is closed. Inflammatory swelling of the lining membrane of the canaliculi may become so pronounced as to obstruct the excretion of the tears; or a foreign body—e.g. an eyelash—may find its way into a canaliculus, most frequently the upper, and not only form a complete barrier to any outflow, but also set up considerable inflammation. The canaliculi may be obstructed by the formation of calculous deposits in their interior.

Excess of tears renders vision indistinct, and constant wiping of the eyes produces considerable irritation, and a certain amount of conjunctivitis develops. Watering and inflammation always increase with exposure to cold winds, but, as a rule, pass off when the weather becomes warm. An examination of the nose should always be made in such cases, for the nasal mucous membrane will often be found to be involved. The nostril on the affected side may be dry and slightly inflamed, or there may be a swollen and œdematous condition of the spongy tissue covering the inferior turbinated bone.

Certain cases which present a well-defined clinical picture have been grouped under the name of "lachrymal catarrh." There is a history of "cold in the head," followed by a sensation as if a foreign body had entered the conjunctival sac, and an itchy feeling in the eyelids is followed by a burning sensation. There is excessive lachrymation, and after a time a slight mucous discharge is seen adhering to the roots of the eyelashes; this may be so abundant as to glue the lids together during sleep. The conjunctiva is injected, and the caruncle and semilunar fold are swollen. When the eyelid is pulled outward the lachrymal papilla is seen to be enlarged, and the punctum is dilated and patulous—very characteristic symptoms. Pressure over the tear-sac usually causes a slight regurgitation of clear watery fluid through the lower canaliculus. There may be slight photophobia. If the patient be exposed to cold or damp, such symptoms may persist for a long time—sometimes better, sometimes worse, but never entirely absent.

After a " watery eye " has persisted for a time a slight swelling is sometimes seen at the inner canthus, over the region of the lachrymal sac. This, at first insignificant, becomes more and more conspicuous as the disease progresses. It is painless, and the skin glides freely over it. When the tumour is pressed upon, its contents pass downward into the nasal auct and the swelling disappears. In a few hours, however, there is a return to the former condition; a fresh accumulation of tears has formed, and by-and-by this, mixed with catarrhal discharge, produces irritation, which at length passes into inflammation of the wall of the sac. As the tumour grows larger a point is reached when, the nasal duct having become blocked, the contents can no longer be pressed downwards into the nose, but regurgitate through the puncta and overflow the eye. The discharge usually consists at first of glairy mucus, like white of egg, streaked with pus; but this after a time becomes turbid and more distinctly purulent. If such a discharge comes into contact with an operation wound most disastrous results follow. In many cases operated on for cataract the eye has been lost through suppuration arising thus, and to operate when the tear-passages are not permeable is simply to court defeat. The discharge irritates the conjunctiva, which becomes more or less inflamed; ophthalmia tarsi frequently co-exists, and the caruncle and semilunar fold are red and swollen. Whenever one eye alone is thus affected, suspicion ought at once to fall upon the lachrymal passages as the cause of the evil.

As the tumour becomes still larger, pressing fails to empty it, because the canaliculi are now blocked as well as the nasal duct. The walls of the sac become relaxed and thinned from the pressure of the accumulating discharge; and a swelling of a bluish-grey colour, of a size varying from a horse-bean to a walnut, fluctuant, and grooved on its anterior surface by the internal palpebral ligament, forms at the inner canthus. This is called a mucocele, and is usually painless, but may produce much disfigurement. It causes constant lachrymation, and patients often complain of dryness of the nostrils on the same side. When catarrh of the tear sac has existed for a long time, periostitis, and then caries, of the lachrymal bone always follows; so that when a probe is passed along the canaliculi it strikes against bare bone. Blenorrhœa of the lachrymal sac is more frequent among women than men, and more especially in those who, like washerwomen, are exposed to considerable alternations of heat and cold. It is also more likely to occur in those whose health is below par, and that is probably why diseases of the tear passages are so common among women who are nursing children. In some instances it is clearly connected with syphilis or tubercle, and in such cases caries of the bones surrounding the nasal duct always occurs at an early stage of the disease. It is sometimes met with in infants, and, being then due to a congenital narrowness of the nasal duct, passes off, as a rule, spontaneously as soon as the tear passages become fully developed.

Acute inflammation of the tear sac is usually a complication of a chronic catarrh, and comes on as a result of exposure to cold. It may, however, also occur after any injury in the neighbourhood of the sac, in the course of affections of the nasal mucous membrane, or after measles and scarlatina. Occasionally infants are born with dacryocystitis, or it comes on within a few weeks of their birth. The onset of the disease is usually marked by shivering and rise of temperature, and there is intense pain in the region of the sac, where the skin becomes red and œdematous. Between the root of the nose and the inner canthus a swelling appears, at first well defined, and so painful that the patient shrinks the moment it is touched. Very soon, however, as the surrounding tissues become involved, it gets more diffuse, and extends to the lids and cheeks; the symptoms of local inflammation are sometimes so severe that the disease has been mistaken for erysipelas, and this error may all the more readily occur when the initial feverishness is great. As the size of the swelling increases, the skin assumes a more angry red, and becomes glistening from the tension to which it is subjected. If not opened by operation, the abscess may burst externally; and if the nasal duct be permeable, the opening will close and the cure be complete; otherwise a fistula will form. In cases which have been long neglected, especially in scrofulous and syphilitic subjects, the surrounding tissues become inflamed, the adjacent bones become carious, and inflammatory swelling and sprouting granulations appear round the fistula. In a few rare cases the abscess may not burst externally, but perforates the lachrymal bone, and its contents escape into the nose.

As has been said, acute dacryocystitis may be mistaken for erysipelas, and the disease may also be confounded with abscess at the root of the canine tooth; but here the internal canthus will be free from tenderness and circumscribed swelling, while palpation of the canine fossa by the mouth will detect a swelling there. In hordeolum or ordinary " stye " the swelling of the eyelids and the parts around may be very great, but palpation will show that the tender spot is not over the tear sac, but at a point on the margin of the eyelids, where in a day or two an abscess will form and burst. In the accurate diagnosis of all tumours, abscesses of the skin, boils, etc., in the neighbourhod of the sac, and likely to be mistaken for inflammation of it, the absence of a history of previous watery eye is of the first importance.

*Treatment.*—The treatment of these conditions varies according to the stage of the disease when the patient comes under observation.

1. *Simple Lachrymation.*—The cause having been determined, the principles of treatment suggest themselves naturally. Careful examination ought to be made of the teeth, eyes, and nose, and any defects remedied. It is most important to correct any error of refraction with suitable spectacles, and any hypertrophy or inflammation of the nasal mucous membrane must be treated. If there be simply congestion of the erectile tissue over the inferior turbinate bone, much relief will be given by painting the nostrils with a solution of cocaine and menthol, or by inhaling carbolic and menthol smelling salts. If the lower punctum be contracted, it ought to be enlarged carefully, by means of a conical dilator; and if the lachrymation persist, a solution of cocaine in adrenalin (1 in 1,000) should be slowly injected along the canaliculus into the lachrymal sac. If there be no obstruction, the fluid will at once pass into the nose; if, however, the lining membrane of the nasal duct be swollen from congestion, the passage of the fluid will be delayed until the adrenalin has had time to constrict the vessels and render the canal pervious. It must be remembered that the use of adrenalin in this way is occasionally followed by acute coryza, which, however, is usually very transient.

2. *Lachrymal Catarrh.*—Astringent lotions, or astringents combined with alkalies, diminish secretion and subdue conjunctival inflammation; and dusting the eyelids with calomel, well dried and finely powdered, often gives marked relief to the sensation of itching and burning heat. Brushing the palpebral conjunctiva with a solution of nitrate of silver (from 2 to 4 per cent., according to the amount of the discharge), or pencilling the lids with a crayon of alum, of sulphate of copper, or even of solid caustic, often aids very materially in restoring to the puncta their natural position and their proper function. When, however, the eversion of the lids is too great to be overcome by such simple means, it is necessary to slit up the canaliculus as far as the lachrymal sac, and so convert the duct into a little gutter along which the tears may escape. The eyelid having been pulled outwards, so as to keep it on the stretch, the blade of a Weber's knife is introduced into the canaliculus and pushed horizontally inwards until its point is felt to strike against the lachrymal bone. The cutting edge ought to be inclined backwards and upwards, and the incision of the canaliculus is completed by simply raising the handle of the knife. For two or three days the lips of the wound must be kept from adhering by passing a probe along their edges. Sometimes the punctum is partially occluded, or there may be an obstruction in the canaliculi, most frequently just where they unite, or at the point where they enter the sac. Such obstacles may be overcome by the use of small probes; but the greatest care is necessary to avoid making a false passage. The natural drainage of the tears having been established, the progress of the case ought to be quite satisfactory, and in most instances nothing more requires to be done. Any defects in the eyes or nose must be remedied, as advised for simple cases.

3. *Catarrh of the Lachrymal Sac and Stricture of the Nasal Duct.*—In the milder cases all that is necessary is to keep the sac empty by pressing on it with the tip of the little finger; and the patient often acquires such dexterity in this that he can do it much more thoroughly than any one else can. In addition, the sac and nasal duct ought to be washed out by alkaline antiseptic solutions by means of a small syringe. If the duct be free from obstruction the fluid passes into the inferior meatus, and, if the patient incline his head forward, flows out of the nostril. These injections ought to be repeated every day, or every second day. The nasal mucous membrane will also require attention, and a similar solution may be sniffed up the nostrils or sent through by means of a syphon douche.

When, however, the contents of the sac cannot be pressed downwards into the nose owing to simple inflammatory thickening of the mucous membrane, Bowman's probes should be used to clear the canaliculi, sac and duct. Some surgeons make a practice of introducing a probe by the superior canaliculus; but, on the whole, it is more convenient and easier to utilise the inferior. The lower eyelid is pulled outwards by the forefinger of the left hand, while with the right the probe, previously bent so as to present a concavity forward (this makes its subsequent passage into the duct easier), is pushed into the punctum, at first perpendicularly, and then, the handle being depressed, horizontally along the canaliculus until it enters the sac and is stopped by the bone at the inner wall. The direction of the probe must now be completely changed in order to reach the nasal duct. While the point is kept firm against the inner wall the handle is raised until it assumes a vertical position. The finger of the left hand ought then to be removed from the lower lid, and, with the thumb or forefinger, the skin of the forehead should be raised and made tense, the passage of the probe into the entrance of the duct being thus facilitated. The probe ought now to be pushed gently, but steadily, downwards, backwards, and a little outwards, following the direction of the duct, until it reaches the nose. Great care must be taken not to tear the mucous membrane and form a false passage; and while firm pressure is often needed to overcome a stricture, anything approaching to violence must be avoided.

When the probe strikes against dead bone, and a firm stricture is also present in the duct, it is necessary to slit up the lower canaliculus and open into the sac, according to the method first proposed by Bowman. In cases where the lower canaliculus is obstructed, the operation may be performed through the superior. A large probe ought now to be passed along the nasal duct. The probe I prefer is that devised by Ziegler, and as the use of all such large probes is very painful I prefer to have the patient under a general anæsthetic, nitrous oxide being, as a rule, sufficient. After the probe has been passed a lead style is introduced; this can be so tapered and curved that it lies in the groove formed by the slit in the canaliculus, and is almost invisible. One can generally remove the style in from 48 hours to a fortnight after the operation, but it can be worn for months without discomfort. It is better, however, for the patient to have it taken out at regular intervals, and the sac washed with an astringent lotion. Suppuration is diminished by the injection of 25 per cent. argyrol solution, but the prolonged use of this or of protargol may be followed by indelible staining of the conjunctiva. When the duct is closed by a bony stricture it will be necessary to perforate the lachrymal bone, and thereby establish a passage directly into the nose.

In addition to local treatment, however, careful attention must be paid to the general health. The strength of patients thus affected is often much run down, and good food and protection from exposure to cold will do a great deal to expedite the progress of a chronic case. Tubercular cases are sometimes bilateral, and in them, as well as in those complicated by syphilis, constitutional treatment is most essential. Anything like very forcible dilatation of the lachrymal duct must here be carefully avoided, as being likely to still further damage the bony walls of the canal, which are, in all likelihood, already extensively diseased.

4. *Acute Dacryocystitis.*—In the rare cases seen quite early the trouble may be arrested by pressing upon the sac to evacuate its contents, prescribing an evaporant lotion, administering a purgative, and applying leeches. As soon, however, as it is quite clear that pus has formed within the tear sac, use should be made of fomentations, and of opiates to relieve pain. Whenever fluctuation can be detected, a free incision ought to be made right into the sac, beginning about the middle of the lower border of the internal palpebral ligament, and enlarged downwards for about half-an-inch in order that the pus may have free exit. The passage ought then to be cleansed by gentle syringing with a warm antiseptic solution, and a probe introduced through the incision into the nasal duct, when, if this channel be successfully opened, the contents of the sac will escape into the nostril and the skin wound will close. In many cases, however, it is most satisfactory not to interfere with the canal until all signs of acute inflammation have disappeared, and then to slit the lower canaliculus, pass a large probe into the nasal duct, and introduce a style, which can be worn as long as may be deemed necessary.

5. *Permanent Enlargement of the Lachrymal Sac, with or without a Fistula.*—In cases of very old standing, where the nasal duct has become obliterated, and there is extensive necrosis of bone, a permanent fistula, situated in the midst of a fungating mass of granulation tissue, remains. In other instances the walls of the sac become thickened and completely inelastic, and in these, even after a free passage has been re-established, lachrymation is very little (if at all) relieved, and an unsightly swelling remains at the inner canthus. In the treatment of all the cases previously described, the aim was to conserve and restore the function of the natural passages; in this group, on the other hand, in order to effect a cure, the natural passages must be completely obliterated. This is best done by Rollet's operation: a general anæsthetic is necessary, and precautions must be taken to prevent blood passing into the pharynx via the nasal duct.

An incision about half an inch in length, curved slightly outwards, is made through skin and subcutaneous tissue down to the bone, beginning at the middle of the lower border of the internal palpebral ligament, which is made more distinct by pulling the lower lid outwards. The lips of the wound are conveniently held apart by a Müller's speculum; and hæmorrhage, which (owing to the irregular distribution of the angular and palpebral branches of the facial artery and vein) is often troublesome, must be checked by seizing any bleeding-point with pressure forceps, and by controlling the general oozing with retractors held firmly against the upper and lower extremities of the incision. A solution of adrenalin and cocaine injected ten minutes before the incision is made is undoubtedly helpful as a hæmostatic. After the field of operation is cleared of blood, the fibrous covering of the sac (which can be readily distinguished by its colour) is cut through, and the sac itself, by means of a rugine, carefully dissected from the lachrymal fossa, caught by forceps, and kept on the stretch while it is gradually freed from its adhesions. Great care must be taken to get thoroughly over the dome, in order to accomplish which it may be necessary to enlarge the incision by dividing the internal palpebral ligament. After it has been completely cleared, the sac, still held firmly by the forceps, is cut through with curved scissors at its junction with the nasal duct (great care being taken all through not to injure the eyeball), and the orifice of the duct is thoroughly scraped with a small Volckman's spoon. One stitch of fine silkworm gut about the middle of the incision will be found sufficient, so that free drainage may take place above and below it. A wet dressing is applied, and, as a rule, left for three days, by the end of which time union has generally taken place. If necessary, the scar may be protected for a few days after that with collodion. The cicatrix is small, and if the incision be made in one of the natural furrows of the skin, it is, in a few weeks, hardly noticeable.

Sometimes, where suppuration has been prolonged, it may be impossible to dissect the sac out owing to the extent of the ulceration in its walls, and it has then to be removed piecemeal with a Volckman's spoon. The cavity should then be packed with gauze, and the granulating surface syringed with antiseptic lotions after this has been removed.

The immediate results of complete extirpation of the lachrymal sac are perfectly satisfactory, for the patient is freed from the disfigurement of the swollen sac, as well as from a continual source of suppurative discharge with all its attendant dangers. The remote results are, as experience has shown, equally good, because abnormal lachrymation occurs only if the eye be exposed to cold winds or other sources of irritation. It may at first seem strange that a constant epiphora should be cured by obliteration of the tear passages; but an explanation is readily to be found in the fact that the lachrymal gland may be regarded as a reserve apparatus, and that, therefore, under normal circumstances, it secretes very little. The natural moisture on the surface of the eyeball is carried away by evaporation, and tears flow only in response to an irritant. An inflamed lachrymal sac is a constant source of irritation, and whenever it is removed the lachrymal gland ceases to secrete abnormally. Moreover, Rollet has shown by his experiments on dogs that ablation of the tear sac is followed by atrophy of the lachrymal gland, and consequently he regards gland and sac as forming part of one system and functionally interdependent.

